# From hepatitis C infection control to elimination: the experience of prisons in Catalonia, Spain between 2020 and 2024

**DOI:** 10.2807/1560-7917.ES.2026.31.44.2600195

**Published:** 2026-11-05

**Authors:** Andrés Marco, Indiana Abdo, Enrique Gil-Tubella, Isabel Barnés, Neus Solé, Elisabet Turu, Group for the control of infections in prisons of Catalonia (GRUMIP), Collaborators, Celia Cols, Laia Escayola, María Jesús Javierre, Efrain Moreno-Valdiris, Ana Ruíz, Concepción Solé, Carlos Touzón, Elisa Vaz

**Affiliations:** 1 Prison Health Programme, Catalan Health Institute, Barcelona, Spain; 2 CIBER of Epidemiology and Public Health (CIBERESP), Carlos III Health Institute, Madrid, Spain; 3 Members of the working group are listed under Collaborators and at the end of the article

**Keywords:** prisons, hepatitis C chronic, public health, illicit drugs, vulnerable populations

## Abstract

**BACKGROUND:**

Prisons are key intervention sites for eliminating hepatitis C virus because people who are incarcerated have a high virus prevalence.

**AIM:**

We aimed to evaluate the effectiveness of a continuous, prison-wide hepatitis C virus elimination programme in Catalonia, Spain.

**METHODS:**

We determined hepatitis C virus prevalence among individuals entering nine prisons (2020–2024), cumulative incidence, and programme effectiveness based on annual incidence and number of direct-acting antiviral (DAA) treatments (2016–2024). Effectiveness was defined as zero incidence or hepatitis C virus RNA < 0.5% (unlikely transmission). Multivariate analysis assessed variables associated with reinfection.

**RESULTS:**

Of 34,088 individuals entering prison, 93.9% were tested (29,826 males; 2,176 females), revealing 370 (1.2%) infections, with a downward trend from 2.2% in 2020 to 0.6% in 2024 (p<0.001). Analysis of 361 infections showed higher frequency in men, Spanish nationals, Barcelona prisons, people who inject drugs, and those living with HIV; 82 were reinfections. Mean time to reinfection was 1,185 ± 164 days, shorter (p = 0.001) in foreign-born individuals (730 ± 290 days); 340 (94.2%) were treated, and 334 (98.2%) sustained virologic response. Incidence remained < 0.5 per 100 person-years from 2020. At 31 December 2024, no viraemia was detected in four of the nine prisons studied.

**CONCLUSION:**

It is possible to eliminate hepatitis C in prisons through universal screening, prompt treatment and reinfection monitoring.

**Key public health message**

**What did you want to address in this study and why?**

We evaluated the effectiveness of a hepatitis C virus (HCV) elimination programme in prisons across Catalonia, Spain, between 2016 and 2024. Addressing HCV in correctional facilities is essential for meeting World Health Organization 2030 elimination goals, as individuals entering prison have a high virus prevalence that heavily impact global viral transmission networks.

**What have we learnt from this study?**

This study demonstrates that hepatitis C micro-elimination in correctional settings is achievable. Through universal HCV screening upon entry to prison and prompt treatment, the programme successfully kept hepatitis C incidence below 0.5 per 100 person-years from 2020 to 2024, achieving complete viral elimination in four of nine facilities by the end of the study period.

**What are the implications of your findings for public health?**

Sustaining these proactive strategies and closely monitoring potential reinfections are critical to maintaining successful public health outcomes over time. By breaking HCV transmission networks, the applied strategies are able to directly improve community health. This intervention provides a scalable model of best practices that can be replicated in other prison systems globally to advance viral elimination.

## Introduction

The global hepatitis C virus (HCV) epidemic disproportionately affects marginalised populations such as people in prison and people who inject drugs (PWID), who represent the largest reservoir of infection and the main drivers of this public health problem. In fact, global modelling estimates published in 2019 show that, if the increased risk of HCV transmission among PWID were eliminated, between 25% and 67% of incident HCV infections in the period between 2018 and 2030 would be avoided, and this proportion rises to 79% in high-income countries [1]. Consequently, eliminating HCV infection among people in prison would be important to elimination efforts in PWID as a whole [2]. To achieve this, the World Health Organization (WHO) has recommended that middle- and high-income countries focus elimination efforts on these priority populations, called micro-elimination, in order to reduce overall HCV infection rates [3].

Prisons provide a suitable environment for intervention as they are high HCV infection settings and some groups in prison, such as undocumented migrants or PWID, are often not covered by conventional care resources. Spain is among the countries with the highest proportion of patients treated for HCV [4]. Although isolated micro-elimination programmes have been successfully implemented in prisons [5-7] and have proven to be cost-effective [8], evaluating continuous, system-wide interventions is essential for providing important progress towards WHO goals for HCV elimination by 2030 [9].

Catalonia, a region of Spain with approximately 8 million inhabitants, has nine prisons located in its territory. The prison population in Catalonia averages around 8,500 people annually [10]. Healthcare for people in prison is managed by the Catalan Health Institute's Prison Health Programme, which ensures that the same level of care is provided as in the general population. In 2002, when the prevalence of HCV infection was at 40%, a prison hepatitis programme was implemented to prevent transmission, diagnose infection, and treat infected individuals with the indicated therapies [11]. Since 2016, all nine Catalan prisons have offered integrated services within a common programme for the elimination of HCV infection: universal and voluntary HCV screening on entry and periodic screening if people in prison engage in risk behaviours; prescription of direct-acting antiviral (DAA) therapy and periodic HCV RNA testing to detect reinfection; use of opioid substitution therapy and needle exchange programs; and educational activities on HCV prevention. These services are centrally organised to avoid disruptions caused by transfers between prisons. Furthermore, upon release, continuity of care and HCV treatment is coordinated with external services through a liaison nurse to ensure continuity of care to the extent possible.

The HCV elimination programme for prisons requires continuous supervision and regular maintenance, and needs to be evaluated periodically. For this reason, it is essential to have reliable data on the prevalence and incidence of HCV, as well as on the risk behaviours of people in prison, the prevention measures adopted, and the provision of treatment [12]. Consequently, while the main challenge is the elimination of the infection, i.e. reducing the incidence to zero, it cannot be the sole criterion for evaluating the effectiveness of these programmes, since the prison population changes daily and incident cases are often newly incarcerated people. Therefore, while maintaining the aim of achieving zero incidence, the programme is considered effective if the incidence rate is maintained below a threshold where transmission remains unlikely [13] – specifically, a threshold defined as an HCV RNA rate in the prison population consistently below 0.5% [14].

The objective of this study is to examine the prevalence of HCV infection in individuals entering any of nine prisons in Catalonia over a period of 5 years, and the cumulative incidence and trends of infection in the prison population since 2016, the year in which treatment with DAAs began in Catalan prisons. In addition, we aimed to evaluate the programme by understanding the evolution of the number of treatments prescribed during the period 2016–2024, determining the status of infection control based on HCV incidence, and assessing complete elimination of viraemia in some of the prisons analysed.

## Methods

### Study design, setting and population

This multicentre, population-based study was designed to examine the prevalence and incidence of HCV infection in people in prison in Catalonia, Spain and evaluate the effectiveness of the programme implemented to achieve elimination.

To study HCV prevalence, people who entered any of the nine penitentiaries in Catalonia between 1 January 2020 and 31 December 2024 were included. Participating centres were located in Barcelona (n = 6), Girona, Lleida and Tarragona. The cumulative incidence and annual trends of HCV infection were also calculated for the entire prison population (both newly admitted and already incarcerated). The effectiveness of the programme was assessed based on the annual incidence rate and the number of DAA treatments prescribed from 2016 until 31 December 2024, the study's end date. The programme was considered effective if HCV incidence was zero or remained below a specific threshold (HCV RNA < 0.5%), where transmission was unlikely.

### Data sources and collection

Sociodemographic data were obtained from the Department of Justice database [10]. Analytical, clinical and therapeutic data were gathered from the health interview conducted during the prison entry assessment or from the shared medical record of Catalonia (HC3) [15], which includes a comprehensive set of web services allowing healthcare professionals to input and consult information on individuals treated in healthcare centres.

The HC3 compiles comprehensive health records from both community and correctional healthcare providers, including data from outpatient visits prior to incarceration, as well as reports of complementary tests, e.g. blood tests, radiological, elastographic and tomographic assessments, and hospital discharge summaries. This regional shared network is accessible to authorised healthcare professionals within the prison system. Through this platform, we reviewed the individuals' medical history outside of prison to verify whether those identified as infected had received prior HCV treatment, achieved a sustained virologic response (SVR), or experienced a recurrence before or during their current imprisonment.

The following variables were used for analysis: date and prison of entry, age, sex assigned at birth, origin (classified as Spanish-born or foreign-born, defined strictly by the individual's country of birth outside of Spain), history of injected drug use, viral RNA (IU/mL), genotype, degree of fibrosis according to transient elastography [16] and HIV infection. Given that the limited sample size of individuals originating from other European Union countries precluded independent analysis, all individuals born outside of Spain were grouped into a single migrant category for statistical analysis. Advanced liver disease was defined as fibrosis stage ≥ 3. Cases with liver cirrhosis were classified according to the Child-Pugh Score. In instances of reinfection, the time between the date of SVR and the new detection of infection was recorded. 

For analysis, correctional facilities were classified based on their primary administrative role and geographic distribution. In the provinces of Girona, Lleida and Tarragona, a single prison per region operates under a mixed regime, housing both individuals in pretrial detention and sentenced individuals. Conversely, facilities in the city of Barcelona are highly specialised, capturing remand (not yet tried) individuals, women, and young people (aged 18–21 years), distinguished in the statistical analysis by the variable 'BCN-prison'. This distinction is critical because pretrial detention centres feature a remarkably high population turnover and substantially shorter lengths of stay, which creates different epidemiological dynamics and a higher potential for HCV transmission compared to centres dedicated exclusively to sentenced individuals.

### Data analysis with main aspects

#### Prevalence

The detection of HCV infection in people who entered any of the nine penitentiary centres was performed using a single-step universal screening method based on reflex testing (automatic confirmation of viraemia via HCV RNA in the same blood sample if serology was positive). This standardised laboratory protocol was consistently maintained over the entire study period. The prevalence, both overall and annually, was calculated for the population screened on entry to the nine prisons studied.

#### Incidence

The cumulative incidence of HCV infection in the entire prison population was calculated based of the detection of active HCV viraemia confirmed via HCV RNA testing, in the last analysis performed on each individual. Once these rates were established, their annual evolution and trends since 2016 were analysed. Operationally, this represents the proportion of people in prison who contracted the infection during annual periods, obtained through quarterly longitudinal analyses. The denominator population only included individuals free of active disease at the beginning of the period considered, confirmed by a documented negative HCV RNA test result. To avoid overestimating the risk, individuals who could not be tested or who were lost to follow-up were also removed from the denominator. It is important to note that, in Catalan prisons, annual screening for HCV and other blood-borne infections is recommended for people who are HCV-negative but have a history of intravenous drug use or engage in high-risk sexual behaviours. In addition, individuals with ongoing risk behaviours who have been treated with DAA and achieved SVR are recommended to undergo HCV screening quarterly to enable early detection of reinfection [2].

#### Direct-acting antivirals

All identified HCV-positive individuals were referred for specialist evaluation and treatment with DAAs. In Catalonia, DAA treatments for all individuals, including those incarcerated, must be approved by the Catalan Ministry of Health and are recorded along with their main personal, clinical, and therapeutic characteristics. These data are restricted but accessible in pseudo-anonymised form. The programme evaluation was based on the evolution of the number of DAA treatments prescribed during the period 2016–2024 in people in prison infected or reinfected with HCV, the maintenance (or failure) of infection control (HCV RNA < 0.5%), and the elimination of viraemia in the population in the prisons analysed.

#### Factors associated with reinfection

Individuals who had previously achieved SVR following treatment and subsequently tested positive for HCV RNA were classified as reinfections. The reinfection date was defined as the midpoint between the last negative and first positive HCV RNA test date. Variables significantly associated with reinfection were studied; those that showed a possible association with reinfection (p < 0.05) were included in the multivariate analysis using a stepwise regression selection method, sequentially removed, and subjected to a likelihood ratio test. The mean time to reinfection was calculated for each individual and according to origin.

#### Statistical analysis

The normality of the data was verified using the Kolmogorov-Smirnov test, which indicated a normal distribution (p > 0.05). The results are presented in absolute numbers with the mean and standard deviation for continuous variables, and percentages and their corresponding 95% confidence intervals (CI) for discrete variables. To study variables associated with reinfection, bivariate and multivariate logistic regression analyses were performed, calculating the adjusted odds ratio (aOR) and its 95% CI. The multivariate model included sex, prison location (Barcelona prison vs. others), country of birth (Spanish-born vs. foreign-born), history of injection drug use, and HIV infection status. Cox regression was used to determine the influence of the variables on the time to reinfection. Kaplan-Meier curves were used to graphically represent the median time to reinfection, using the log-rank test as the method for comparing groups.

## Results

A total of 34,088 people entered prison in the study period, of whom 32,002 (93.9%) agreed to be screened for HCV. The mean age of those admitted was 38.4 years (standard deviation (SD): ± 6.3; range: 18–81), 92.4% were male (n = 31,497; with n = 2,591 female), and 47.8% (n = 16,294) were foreign-born individuals, with the majority originating from North Africa and Central and South America. A detailed geographical breakdown of individual countries and specific percentages is provided in Supplementary Table S1. Active infections were detected in 370 people in prison (1.2%).

### Characteristics of people in prison with hepatitis C infection

Of the 370 with HCV infection, 361 (97.6%) were included in our analysis. Nine (2.4%) were excluded because they were released from prison or declined diagnostic testing. Of those studied, 270 (74.8%) were untreated and 82 (22.7%) were post-SVR. In the remaining nine (2.5%), the infection had been previously treated but the SVR status was not recorded, and it was not known whether the treatment had been discontinued, had failed, or whether these were reinfections.

The mean age of the 361 people analysed was 45.3 years (SD): +/− 9.3; range: 18–80 years); 331 (91.7%) were male, and 30 (8.3%) were female. Additionally, 150 (41.6%) were foreign-born, 237 (65.7%) had a history of injected drug use, and 99 (27.4%) were HIV-infected. The majority (n = 225, 63%) were admitted to pretrial detention centres in Barcelona. Fifty (13.9%) presented with advanced liver disease and nine (2.5%) with cirrhosis, with a Child-Pugh score of ‘B’ or ‘C’. 

There were significant changes in the number of individuals admitted during the study period because of the COVID-19 pandemic, which resulted in fewer admissions (trend p = 0.010); however, the epidemiological characteristics of the study population did not vary significantly over the 5 years analysed. The detailed annual descriptive breakdown of these demographic and clinical variables is provided in Supplementary Table S2.

A total of 340 people (94.2% of those studied, and 91.9% of those in whom HCV was detected) were treated during their first month in prison. Twenty-one people did not receive treatment, because 19 were released within a very short period and two declined treatment. An overview of the study population screened and treated is shown in Figure 1.

**Figure 1:**
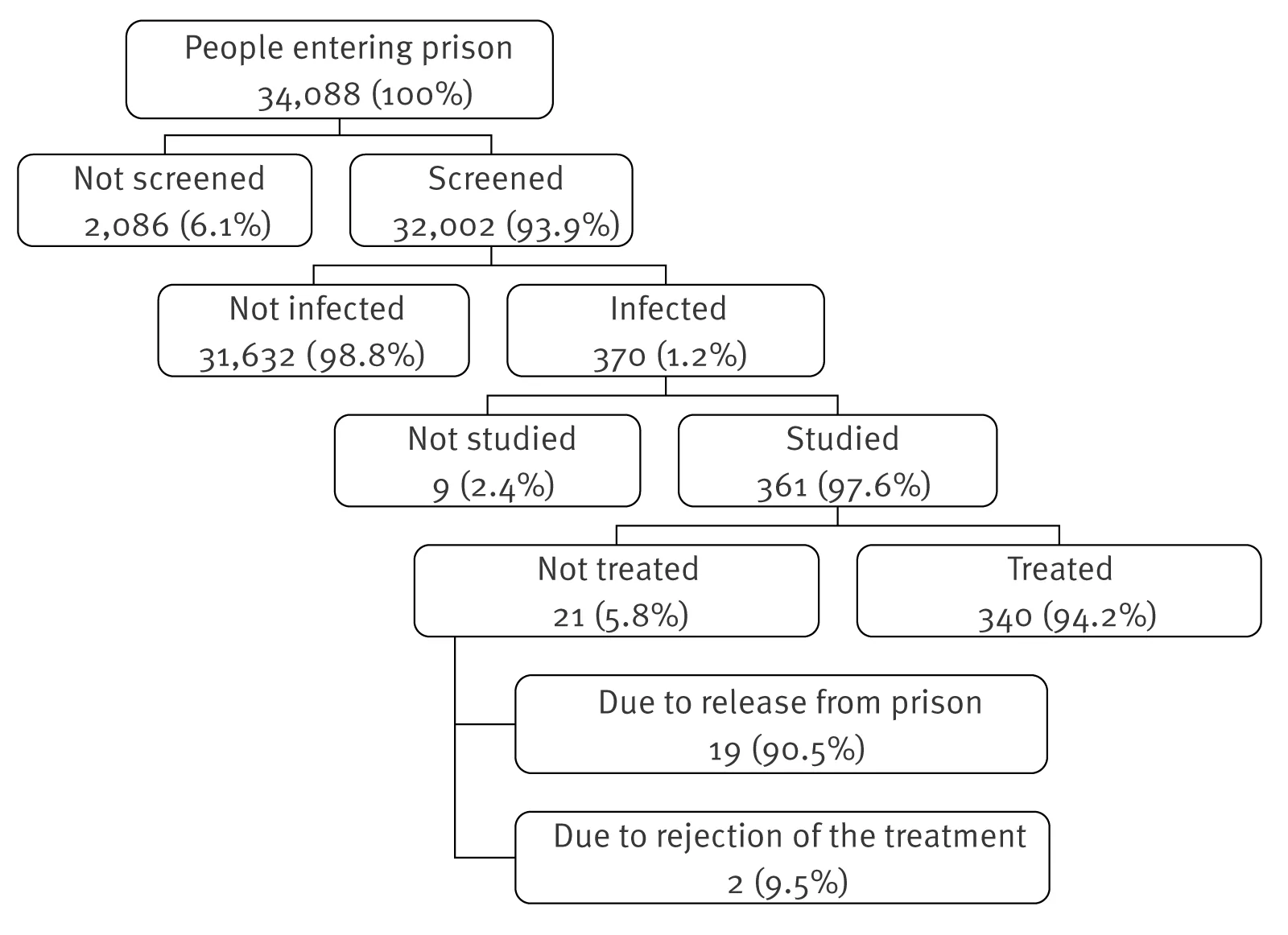
Overview of the study population screened and treated for hepatitis C infection at entry to prison, Catalonia, Spain, 2020–2024 (n = 32,002 screened)

The study encompasses all nine correctional facilities in Catalonia, Spain. 'Treated' refers to individuals who received direct-acting antiviral (DAA) therapy.

There were no differences in the proportion of people entering prison who were screened over time, but the rate of infections detected fell by 72.7% during the 5-year period, from 2.2% in 2020 to 0.6% in 2024 (p < 0.001) (Figure 2).

**Figure 2:**
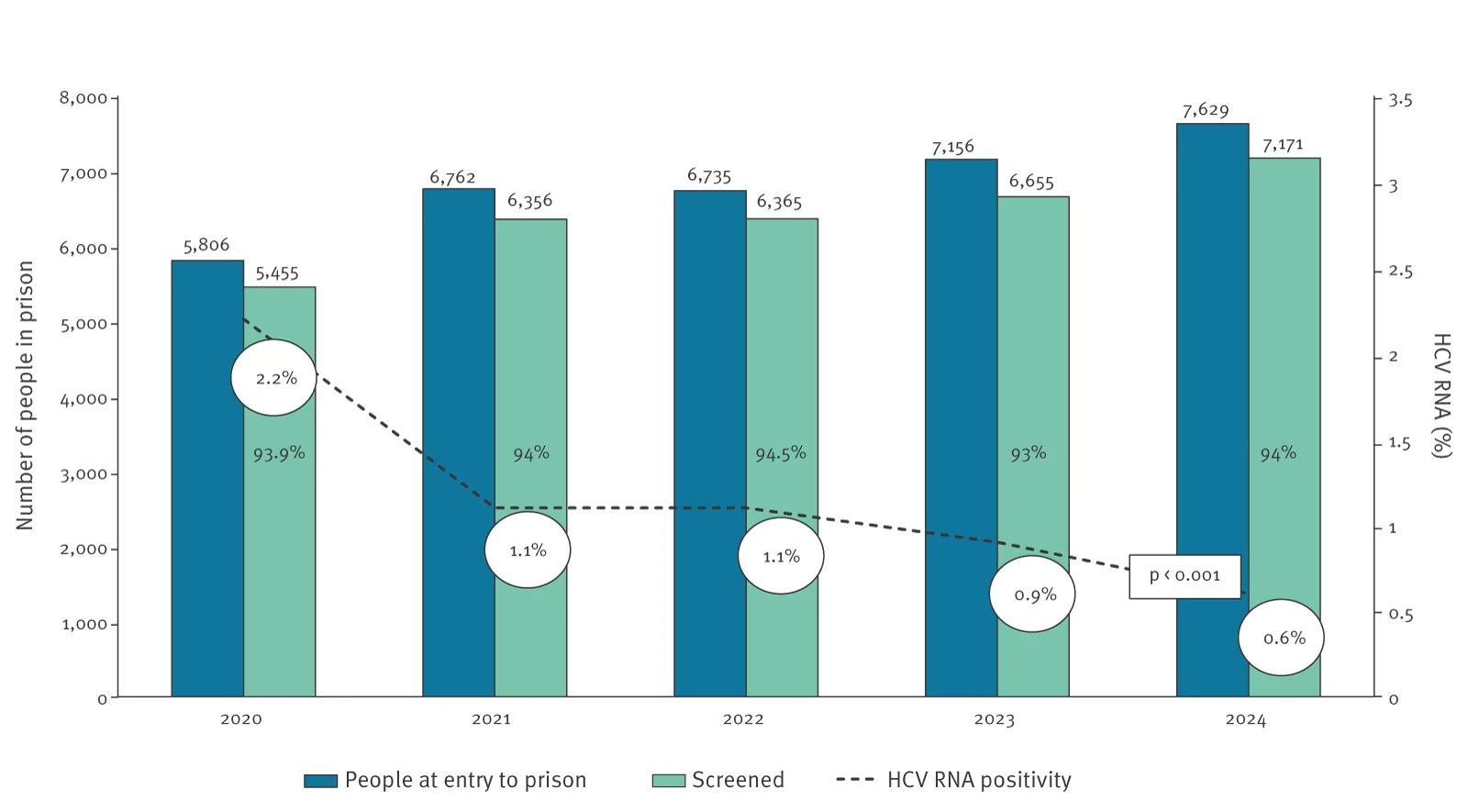
Hepatitis C screening and viraemia in people at entry to prison, Catalonia, Spain, 2020–2024 (n = 34,088)

Of the 34,088 total admissions, 32,002 (93.9%) individuals underwent screening. Annual positive cases supporting the percentages shown were: 2020 (n = 120), 2021 (n = 70), 2022 (n = 70), 2023 (n = 60), and 2024 (n = 43). See Supplementary Table S2 for the detailed annual descriptive breakdown of the population characteristics.

### Determinants of hepatitis C virus reinfection at entry to prison

In the 82 reinfected individuals, the variables associated with reinfection were: (i) male sex (24.5% reinfections in men vs 3.3% in women); (ii) having been admitted to pre-trial detention centres for adults or young people (18–21 years) in Barcelona (27.1% vs 15.4% in those admitted outside the city); (iii) being Spanish-born (26.5% vs 17.3% in foreign-born); (iv) a history of injected drug use (33.3% in PWID vs 2.4% in non-PWID); and (v) HIV infection (47.5% vs 13.5% in non-HIV infected). The odds ratio (OR) of reinfection and its corresponding CI are presented in the Table, along with the statistical significance of the variables associated with reinfection. The median time to reinfection was 1,185 ± 164 days and was significantly shorter (p = 0.001) in foreigners (730 ± 290 days) than in those Spanish-born (1,640 ± 228 days) (Figure 3). There were no significant differences in time to reinfection with regard to other variables studied: age (p = 0.29), type of prison (p = 0.49), PWID status (p = 0.27) or HIV infection (p = 0.48).

**Table:** Determinants of hepatitis C reinfection in people at entry to prison, Catalonia, Spain, 2020–2024 (n = 361)

Variable	Bivariate analysis							Multivariate analysis		
	Reinfected				p value	OR	95% CI	p value	aOR	95% CI
	Yes		No							
	n	%	n	%						
**Sex**										
Men	81	24.5	250	75.5	0.001	9.3	1.26–70.06	0.02	11.38	1.37–94.47
Women^a^	1	3.3	29	96.7		Ref.				
**BCN-prison** ^b^										
Yes	61	27.1	164	72.9	0.004	2.03	1.18–3.58	0.02	2.08	1.08–4.00
No^a^	21	15.4	115	84.6						
**Spanish-born participants**										
Yes	56	26.5	155	73.5	0.01	1.72	1.02–2.90	0.02	2.05	1.11–3.78
No^a^	26	17.3	124	82.7						
**IDU history**										
Yes	79	33.3	158	66.6	< 0.001	20.17	6.21–65.4	< 0.001	15.53	4.65–51.91
No^a^	3	2.4	121	97.6						
**HIV infection**										
Yes	47	47.5	52	52.5	< 0.001	5.86	3.44–9.97	< 0.001	4.32	2.38–7.83
No^a^	35	13.4	227	86.6						

aOR: adjusted odds ratio; CI: confidence interval; IDU: injection drug use; OR: odds ratio; Ref.: reference.

^a^ Reference group: the multivariate analysis was mutually adjusted for all variables listed in the table (sex, prison location, country of birth, injection drug use history, and HIV infection).

^b ^BCN-prison: prisons for detainees, not yet tried, of men, women and young people (18–21 years old) in the city of Barcelona.

**Figure 3:**
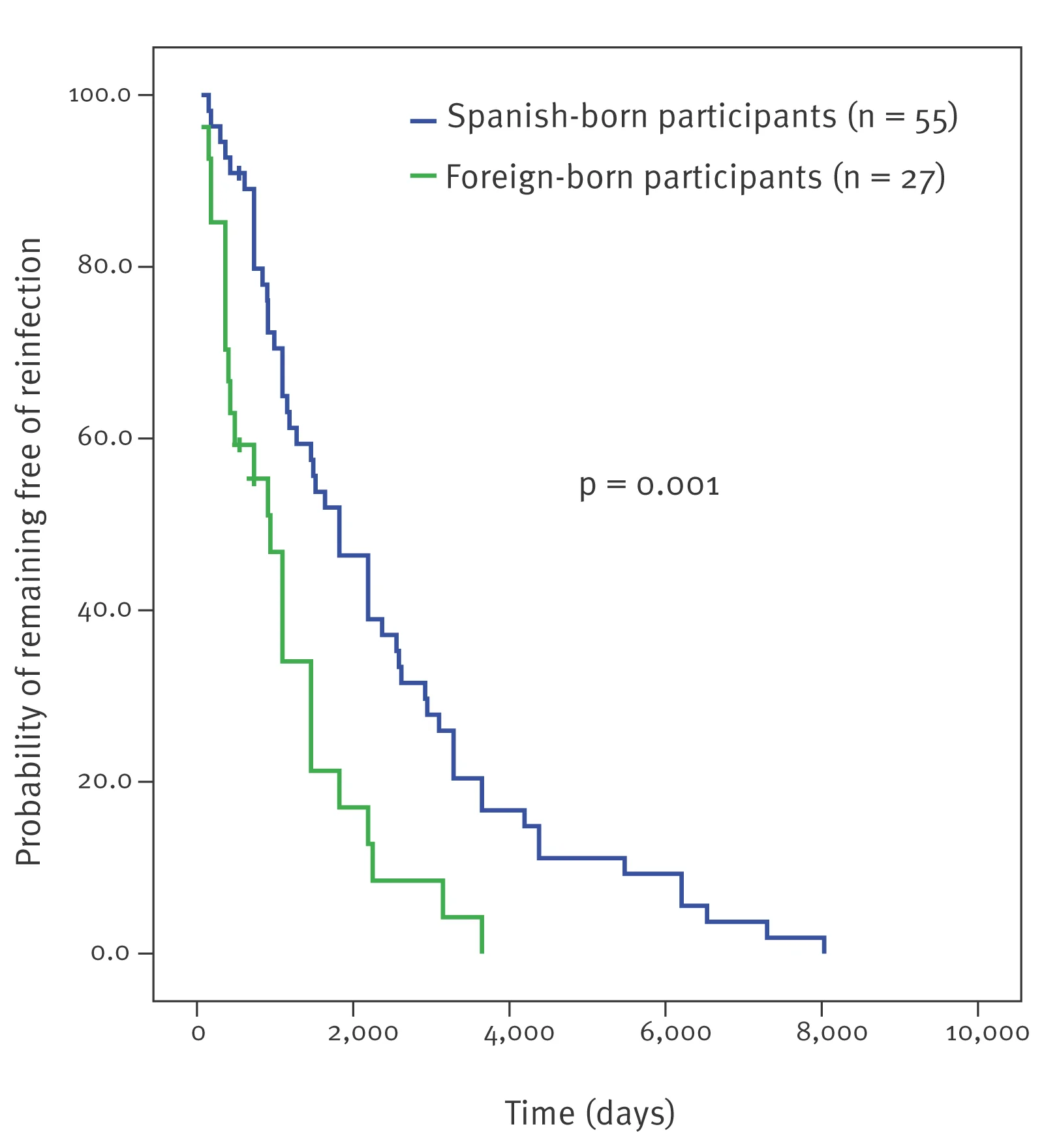
Probability of hepatitis C reinfection among people in prison, Catalonia, Spain, 2020–2024 (n = 82)

The blue line represents Spanish-born participants (n = 55) and the green line represents foreign-born participants (n = 27). Log-rank test: p < 0.001.

From 1 January 2020 to 31 December 2024, 1,335 DAA treatments were prescribed. During this period, the incidence of HCV infection fell from 10.1% in 2016 to 0.3% in the second half of 2020; since then, it has remained below 0.5%. By the end of the study period, no viraemia was detected at four of the nine prisons. At the other five, HCV RNA was identified in 28 people: 10 who had already started treatment, two who declined, one whose treatment had been postponed because of ongoing tuberculosis treatment, and 15 people who recently entered under study and awaiting DAA prescription. The evolution of the number of treatments, the trend in HCV infection, and the number of prisons with undetectable viraemia over the years are shown in Figure 4. 

**Figure 4:**
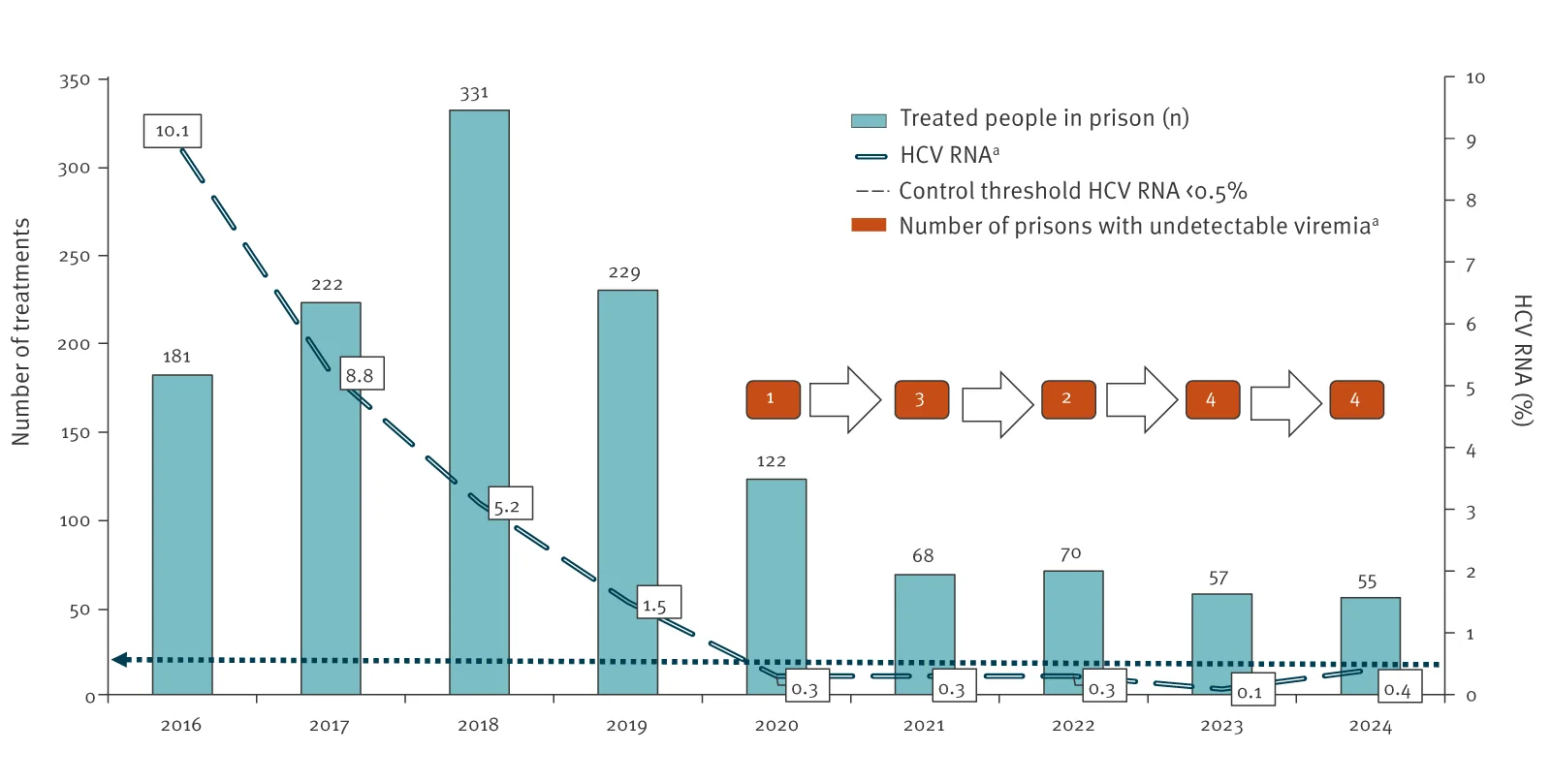
Number of direct-acting antiviral treatments and trend of hepatitis C virus infection in people in prison, Catalonia, Spain, 2016–2024 (n =1,335)

^a^ Data from 31 December of each year analysed.

All treatments administered throughout the study period (2016–2024) consisted of direct-acting antivirals (DAAs).

Regarding the last 5 years analysed (2020–2024), 372 DAA treatments were prescribed for 352 people in prison (91.4% in those infected at entry and 8.6% in those already incarcerated). Ninety-four (26.7%) treatments were prescribed for reinfected individuals. The effectiveness of DAA treatment was 98.4%; only six (1.6%) did not present initial SVR and required retreatment. This sharp decline and subsequent stabilisation of treatments during this later phase reflects the depletion of the infected pool following the intensive historical scale-up between 2016 and 2019, rather than a reduction in screening efforts. The number of treatments administered to people in prison, both infected and reinfected with HCV, is displayed in Figure 5, along with the trend.

**Figure 5:**
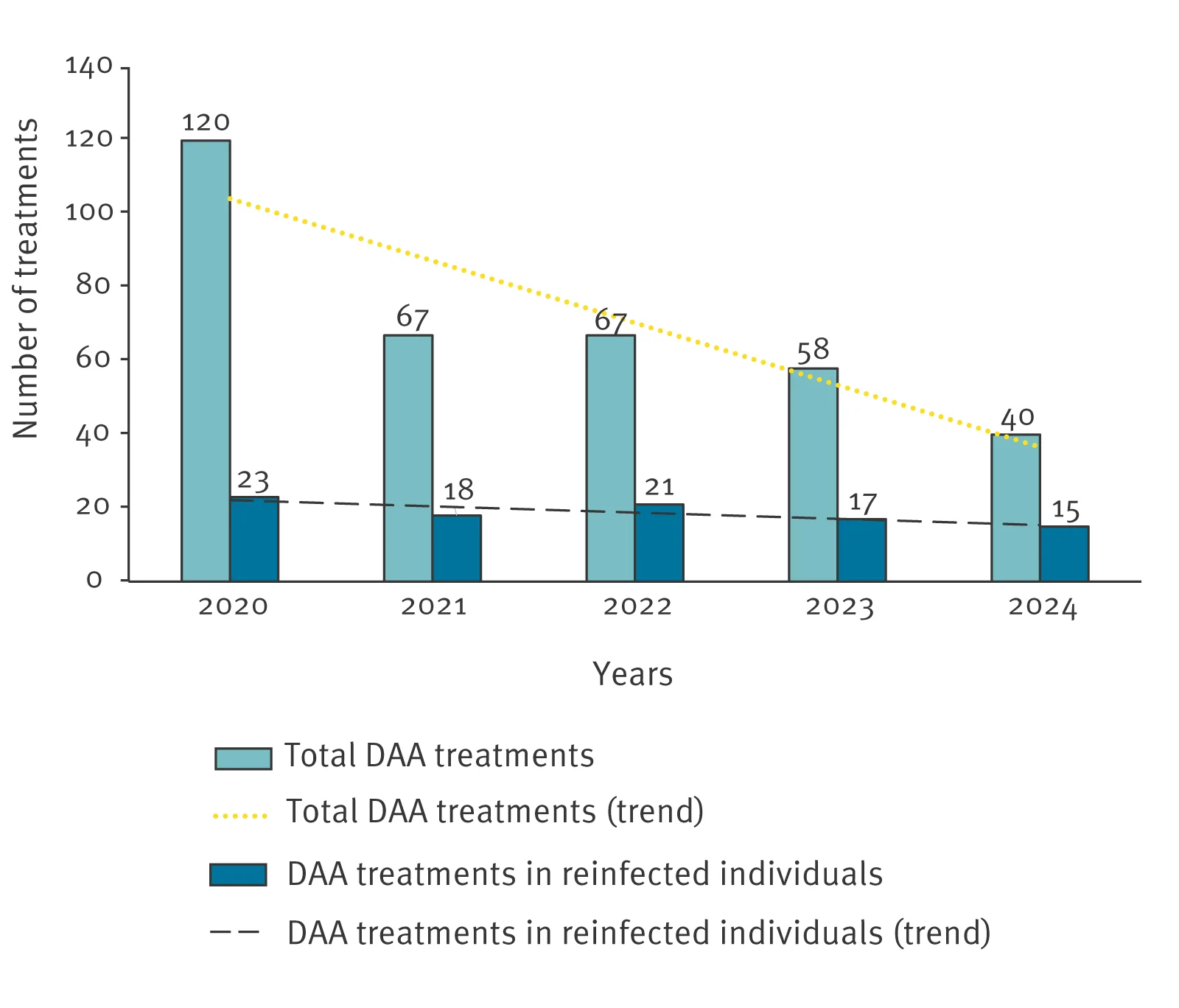
Distribution and trend of direct-acting antiviral treatments administered to people in prison, Catalonia, Spain, 2020–2024 (n = 352)

DAA: direct-acting antiviral.

Treatments strictly refer to therapeutic regimens with DAAs. The values at the top of the bars indicate the exact number (n) of treatment courses prescribed each year: the light blue bars represent total DAA treatments (ranging from 120 in 2020 to 40 in 2024), and the dark blue bars represent DAA treatments specifically administered to reinfected individuals (ranging from 23 in 2020 to 15 in 2024). The dashed lines represent the respective statistical trendlines over the analysed period.

## Discussion

In this study, the mean HCV viraemia rate among people entering prison in Catalonia, Spain between 2020 and 2024 was 1.2%. This figure was obtained by screening 93.9% of the 34,088 incarcerations – an unusually high screening rate [17,18], close to the 95.9% reported by Halford et al. in a British prison project [19]. The viraemia rate detected was higher than that of the general Spanish population (0.15% in 2022) [20] but substantially lower than in prison populations in other countries [17-19,21]. However, it is difficult to perform this latter comparison with total confidence, since only 35% of national hepatitis programmes worldwide include actions for populations in prison [22]. Furthermore, when screenings are available, they are not always performed upon prison entry, are often not universal, and sometimes measure serology but not viraemia. In fact, a recent meta-analysis concluded that data on infection in people in prison are too heterogeneous to establish a reliable, pooled prevalence [23].

Our analysis demonstrates that study of the prison context is essential for discovering undiagnosed individuals, with an estimated 29% undiagnosed individuals in the general Spanish population and 78.6% worldwide [24]. Notably, many of these infections (65.7%) were detected in PWID, a key group for the elimination of HCV infection. It has been suggested that any intervention that can reduce HCV transmission among PWID will have a positive effect on reducing HCV incidence at the national level [25]. However, the implementation of harm reduction programmes and the current levels of diagnostic testing and anti-HCV treatment in this population, particularly in the prison setting, are not sufficient to achieve national HCV elimination goals [22]. In the 5 years analysed, viraemia in incarcerated individuals decreased by 72.7%, in line with the decline in HCV infection observed in Spain [20]. Similar declines in viraemia have been observed in prison studies in other countries [26,27], mainly as a result of the expansion of treatment and, to a lesser extent, the decrease in HCV prevalence in prisons [21]. In Spain, in the last decade, more than 172,000 patients have been treated and cured of hepatitis C [4]. In this same period, 1,335 treatments were prescribed in Catalan prisons. The 352 people in prison treated between 2020 and 2024 and analysed in the present study had a particularly high SVR rate – 98.5%. This is possibly because of the high level of coordination between the prisons involved in the programme, which avoided losses resulting from transfers between prisons, and the role of the liaison nurse, which reduced losses caused by release, a problem cited in previous research [28].

Although treatment with DAAs is a powerful tool for preventing HCV transmission in the prison setting, pharmacological treatment alone may not be sufficient to eliminate reinfections among specific high-risk subgroups, particularly people living with HIV [29]. In our study, 94 treatments (26.7%) were administered to infected individuals who had previously received treatment and achieved an SVR. These were considered reinfections. The actual number could be even higher, as there were nine (2.5%) in which the infection had been treated, but SVR status was not recorded, and it was unknown whether treatment had been discontinued, had failed or were indeed reinfections. These situations were more frequent among people living with HIV, men, Spanish nationals, and people in prison in the city of Barcelona, probably because risk behaviours are more common in large cities. However, the number of reinfections was small, especially in some groups, and therefore these results should be interpreted with caution. Interestingly, the time to reinfection was significantly shorter among foreign-born vs Spanish-born individuals. In Catalonia, ca 40% of people living with HIV are individuals with a history of migration [30] coming from HCV-endemic areas such as Eastern Europe. Overall, people with a history of migration they are 3.3 times less likely to have been screened for HCV and 2.6 times less likely to have started anti-HCV treatment than those born in Spain [31]. In a study of active PWID treated with DAAs at a harm reduction centre in Barcelona, Spain, ​​reinfection rates were very high (31/100 person/years), and 42.9% of those reinfected were foreign-born, and many of them formerly incarcerated individuals [32]. People with a history of migration are extremely vulnerable, and it is not surprising that reinfection occurs earlier in this group. In addition to their use of injected drugs, they face difficulties because of their migrant status and limitations in legal, health and socioeconomic conditions that increase their risk of infection. Furthermore, because these individuals generally use conventional healthcare resources less frequently, the information in their electronic health records (EHRs) may be less complete. Consequently, active monitoring and continuity of care become even more challenging. The success of micro-elimination programmes in HCV-infected PWID living in highly vulnerable social conditions depends on the capacity to combine increased access to treatment with the use of harm reduction programmes, behavioural interventions, and improvements in welfare provision. Detecting and treating reinfected individuals is also crucial and should be a primary objective for facilities with the closest contact with this population, such as drug treatment centres or prisons.

Regarding infection control, our study highlights that HCV viraemia was below 0.5% in all prisons analysed over the 5-year period and, at 31 December 2024, there was no viraemia detected in four of nine prisons included. To our knowledge, this is one of the few studies to report continuous monitoring of HCV viraemia across all prisons within a geographical region. This was possible because of the thorough detection of HCV-infected or reinfected individuals and the rapid provision of treatment, but also due to the characteristics of the programme, which is universal (applied to all people in prison), homogeneous (applied in the same way in all nine prisons), and equitable (providing care comparable to that received outside prison). In our opinion, eliminating HCV in the prison environment requires implementing programmes with intersectoral activities that are interconnected and integrated within a national plan. Isolated micro-elimination projects are commendable and offer individual benefits, but their impact on reducing HCV incidence at the community level is likely to be limited.

This study has several limitations. Firstly, one limitation of our study is that we could not determine the exact number of individuals who experienced multiple incarcerations during the study period. Consequently, a single individual entering and exiting the prison system multiple times could be overrepresented in certain longitudinal trends. Secondly, some of the information was obtained from the HC3 database, which collects much, but not all, of the health information of residents in Catalonia; consequently, these records may be less complete for highly vulnerable subgroups who utilise conventional healthcare resources less frequently. This information is less complete in groups such as PWID and/or foreign-born individuals, although the information collected on entry proved to be unrecorded in only 2.5% of cases. Thirdly, HCV recurrence post-SVR was interpreted as reinfection and not as late relapse; this definition might be questioned, although it is generally accepted that with current DAA treatment the risk of recurrence associated with late relapse is very low [33]. Fourthly, information regarding risk practices among those reinfected is lacking. By the time the data analysis was carried out, many of the people in prison had been released and could not be interviewed. Much of this information was also absent from the medical records, since people in Catalan prisons who participate in programmes such as the distribution of needles and syringes are not identified.

## Conclusion

HCV elimination programmes in prisons must be sustained over time and evaluated periodically. It is possible to eliminate of HCV infection in these institutions if universal screening is maintained, treatment is administered promptly and potential re-infections are monitored. This programme has shown excellent results and could serve as an example of good practice and a model for other countries.

## Data Availability

The individual-level data analysed in this study are subject to ethical and legal restrictions due to patient confidentiality and data protection regulations governing routinely collected health data within the Catalan penitentiary system and the HC3 database. Consequently, data are not publicly available but may be released in pseudo-anonymised form upon reasonable request, subject to approval by the relevant health authorities and the corresponding ethical review board.

## Supplementary Data

357000 Supplement
