# Supplementary material for: From hepatitis C infection control to elimination: the experience of prisons in Catalonia, Spain between 2020 and 2024
Source: Euro Surveill. 2026 Nov 5;31(44):2600195. doi: 10.2807/1560-7917.ES.2026.31.44.2600195 (PMC13743898; doi:10.2807/1560-7917.ES.2026.31.44.2600195)
Supplement: Supplementary file 1 [file 26-00195_MARCO_Supplement.pdf]

Supplementary Material

This supplementary material is hosted by Eurosurveillance as supporting information alongside the article "From hepatitis C infection control to elimination; the experience of prisons in Catalonia, Spain between 2020 and 2024"· on behalf of the authors who remain responsible for the accuracy and appropriateness of the content. The same standards for ethics, copyright, attributions and permissions as for the article apply. Eurosurveillance is not responsible for the maintenance of any links or email addresses provided therein.

Supplementary Table S1. Geographical distribution of foreign-born individuals admitted to Catalan prisons (2020–2024)

	Years					p-value
	**2020**	**2021**	**2022**	**2023**	**2024**	
**Total admissions**, n	5,806	6,762	6,735	7,156	7629	0.01
**Individuals screened**, n (%)	5,455 (93.9)	6,356 (94.0)	6,365 (94.5)	6,655 (93.0)	7,171 (94.0)	0.32
**Mean age** (years)	44.9	45.4	44.6	45.8	45.6	0.31
**Sex,** n (%)						0.42
• Male	5,336 (91.9)	6,235 (92.2)	6,156 (91.4)	6,512 (91.0)	7,003 (91.8)	
• Female	470 (8.1)	527 (7.8)	579 (8.6)	644 (9.0)	626 (8.2)	
**Foreing-born individuals,** n (%)						0.33
• Yes	2,346 (40.4)	2,845 (42.1)	2,787 (41.4)	2,999 (41.9)	3,191 (41.8)	
• No	3,460 (59.6)	3,917 (57.9)	3,948 (58.6)	4,157 (58.1)	4,438 (58.2)	
**PWID**, n (%)						0.48
• Yes	3,787 (65.2)	4,465 (65.7)	4,487 (66.6)	4,684 (65.5)	4,995 (65.5)	
• No	2,019 (34.8)	2,327 (34.3)	2,248 (33.4)	2,472 (34.5)	2,634 (34.5)	
**HIV infection,** n (%)						0.99
• Yes	1,585 (27.3)	1,859 (27.5)	1,838 (27.3)	1,961 (27.4)	2,098 (27.5)	
• No	4,221 (72.7)	4,903 (72.5)	4,897 (72.7)	5,195 (72.6)	5,531 (72.5)	

PWID: People Who Inject Drugs

Supplementary Table S2. Baseline demographic and epidemiological characteristics of the overall population entering prison (Catalonia, Spain), 2020-2024 (n=34,188)

Region of Origin	Main Representative Countries Percentage	%
**North Africa**	Morocco, Algeria, Tunisia, Libya, and Mauritania	38.1
**Central and South America**	Diverse nations	32.7
**Eastern part of the WHO European Region**	Georgia, Romania, Armenia, Russia, Ukraine, Lithuania, and Poland	14.9
**Other Areas**	Other less represented geographical regions	14.3
